# Cystatin C as a potential therapeutic mediator against Parkinson’s disease via VEGF-induced angiogenesis and enhanced neuronal autophagy in neurovascular units

**DOI:** 10.1038/cddis.2017.240

**Published:** 2017-06-01

**Authors:** Jing Zou, Zhaoyu Chen, Xiaobo Wei, Zhigang Chen, Yongmei Fu, Xiaoyan Yang, Dan Chen, Rui Wang, Peter Jenner, Jia-Hong Lu, Min Li, Zhuohua Zhang, Beisha Tang, Kunlin Jin, Qing Wang

**Affiliations:** 1 Department of Neurology, The Third Affiliated Hospital of Sun Yat-Sen University, Guangzhou, Guangdong 510630, China; 2 Department of Emergency, The Third Affiliated Hospital of Sun Yat-Sen University, Guangzhou, Guangdong 510630, China; 3 Neurodegenerative Diseases Research Group, Faculty of Health Sciences and Medicine, King’s College, London SE1 1UL, UK; 4 State Key Laboratory of Quality Research in Chinese Medicine, Institute of Chinese Medical Sciences, University of Macau, Taipa, Macau, China; 5 School of Chinese Medicine, Hong Kong Baptist University, Kowloon Tong, Hong Kong, China; 6 Department of Neurology, Xiangya School of Medicine and The State Key Laboratory of Medical Genetics, Central South University, Changsha, Hunan 410078, China; 7 Department of Pharmacology and Neuroscience, University of North Texas Health Science Center, Fort Worth, TX 76107, USA

## Abstract

Cystatin C (CYS C, *Cst3*) is an endogenous cysteine protease inhibitor that plays neuroprotective roles in neurodegenerative diseases. We aimed to explore the association of CYS C with Parkinson’s disease (PD) models and investigate its involvement in the role of neurovascular units (NVUs) in PD neuro-pathogenesis. We used A53T *α*-synuclein (SNCA) transgenic mice and 6-hydroxydopamine-lesioned DAergic PC12 cells as experimental PD models to investigate the mechanisms behind this association. The injections of CYS C were administered to the right substantia nigra (SN) of A53T SNCA transgenic mice to measure the effects of CYS C in transgenic A53T SNCA mice. To explore the angiogenesis *in vivo* and *in vitro*, we used the chick embryo chorioallantoic membrane (CAM) assay and tube formation (TF) assay. We found that CYS C has a neuroprotective effect in this *in vivo* PD model. We observed increased VEGF, NURR1 and autophagy markers LC3B and decreased SNCA and apoptosis marker cleaved CASP3 in different brain regions of CYS C-treated A53T SNCA transgenic mice. *In vitro*, we observed that CYS C-induced VEGF, a secreted protein, attenuated 6-OHDA-lesioned DAergic PC12 cell degeneration by regulating *p-PKC-α/p-ERK1/2-Nurr1* signaling and inducing autophagy. VEGF-mediated angiogenesis was markedly enhanced in the conditioned media of 6-OHDA-lesioned PC12 cells with CYS C-overexpression, whereas blockage of autophagy in CYS C-overexpressing PC12 cells significantly downregulated VEGF expression and the associated angiogenesis. Our data indicate that CYS C displays dual neuronal–vascular functions, promoting PC12 cell survival and angiogenesis via regulating the level of secreted VEGF in NVUs. Our study provides evidence that may aid in the development of an alternative approach for the treatment of PD through modulation of CYS C-mediated neuronal-vascular pathways.

Neural cells and vascular cells form a functionally integrated network that is collectively termed the neurovascular units (NVUs), which regulate important pathological functions in neurodegenerative diseases such as Alzheimer’s disease (AD) and Parkinson’s diseases (PD).^1, 2^ Several lines of evidence indicate that NVUs disruption, especially abnormal neuronal-vascular relationships, play an important role in PD pathogenesis.^3, 4^ In the NVUs, some secreted molecules such as vascular endothelial growth factors (VEGFs) are key components that not only mediate neuronal survival but also maintain vascular homeostasis and promote angiogenesis.^5, 6, 7^ In PD patients, increased VEGF in the CSF is associated with blood–brain barrier (BBB) dysfunction and neural degeneration.^8^ Several lines of evidence have also indicated that the secreted molecule VEGF could regulate angiogenesis and promote neuronal survival.^9, 10, 11^ On the other hand, recent studies have shown that neuronal events such as autophagy could regulate the cerebral microenvironment, leading to disruption of the NVUs.^12^ Therefore, the neuronal–vascular relationship is critical for cerebral functions in aging-related diseases such as PD.

Cystatin C (CYS C), a secreted cysteine inhibitor encoded by the CST3 (*Cst3*) gene, is a 13-kDa protein that consists of 120 amino acids encoded by a 7.3-kb gene located on chromosome 20.^13, 14^ It is commonly used as a biomarker of renal function and is a strong predictor of cardiovascular events and cerebral ischemia.^15^ Moreover, recent studies have shown that CYS C has a neuroprotective role in diseases, such as AD, amyotrophic lateral sclerosis (ALS) and subarachnoid hemorrhage (SAH), by inducing cellular autophagy.^16, 17^ CYS C also exerts its function by regulating vascular remodeling and integrity.^18^ Taken together, these findings strongly suggest that CYS C could be a novel secreted protein that induces cellular autophagy and induces angiogenesis in the cerebral microenvironment. Whether there is an association between CYS C and VEGF and how this relationship influences neurodegenerative diseases such as PD is an interesting topic to explore.

In the present study, we therefore sought to determine: (1) how VEGF/NURR1 levels and autophagy change in the brain of CYS C-treated A53T *α*-synuclein (SNCA) mice, an *in vivo* PD model; (2) in an *in vitro* study, whether CYS C exerts neuronal–vascular dual functions in promoting DAergic PC12 cell survival and angiogenesis via regulating the secreted protein VEGF in NVUs; (3) how CYS C-mediated enhanced DAergic neuronal autophagy influences VEGF and VEGF-induced angiogenesis in NVUs.

## Results

### *In vivo* study: increased VEGF, NURR1 and autophagy, and decreased SNCA in CYS C-treated A53T SNCA mice

**Figure 1:**
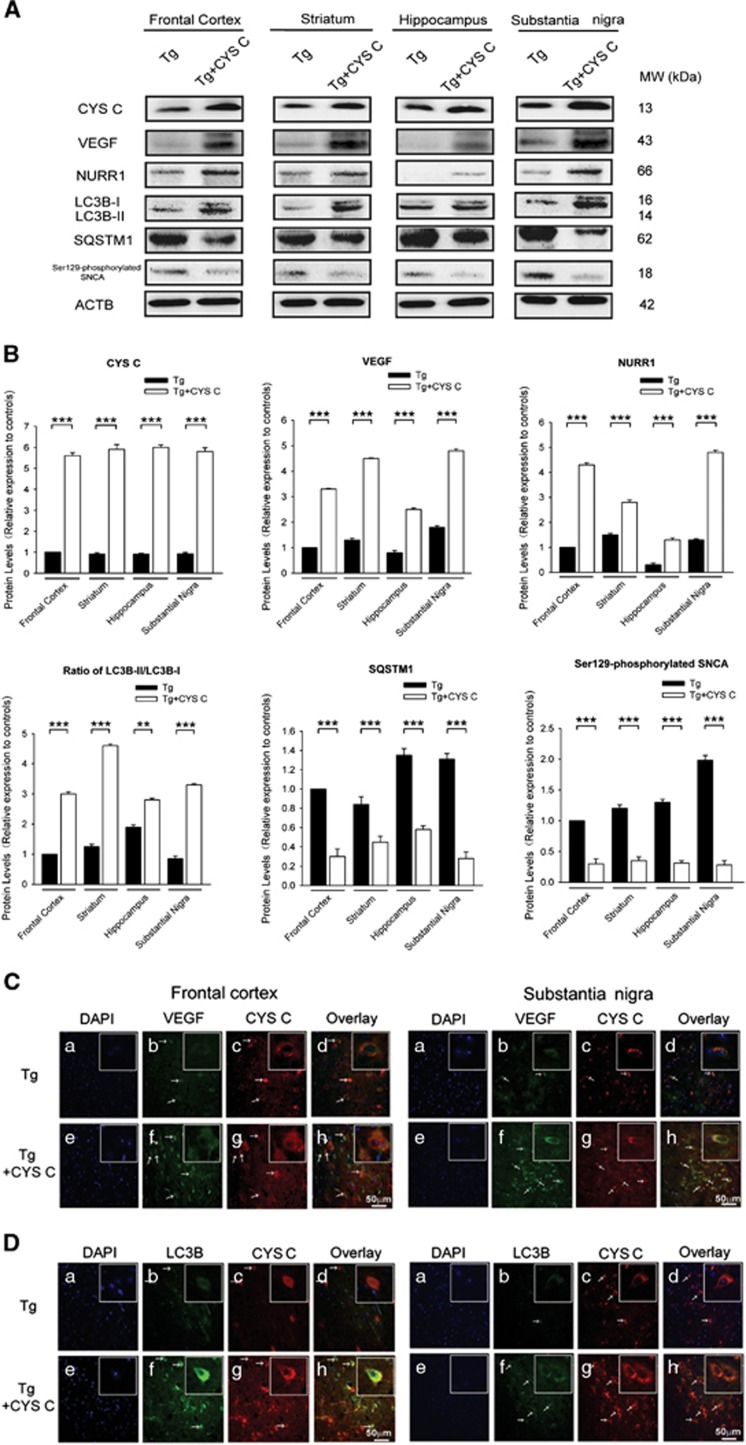

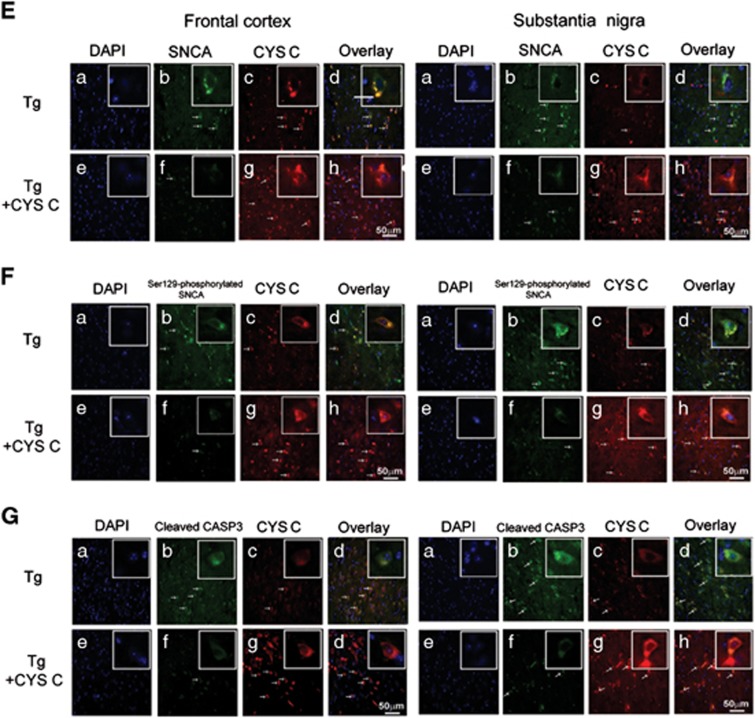
CYS C displays a neuroprotective effect in an *in vivo* PD model. (**A** and **B**) The protein levels in different brain regions in an *in vivo* PD model. Tg mice were generated to express the mutant human A53T SNCA and used as the *in vivo* PD model. The levels of CYS C, VEGF, NURR1, LC3 B-I/II, SQSTM1 and Ser129-phosphorylated SNCA in four brain regions (frontal cortex, striatum, hippocampus, and substantia nigra) of 6-month-old Tg mice and cystatin C-treated Tg mice, were measured. The bar chart (**B**) shows the relative quantification of the specific protein levels compared with that of ACTB. The data are expressed as the relative ratios of the blank group, which was set to 1.0, and are expressed as the mean±S.E.M. of five independent experiments. ***P<0.001. (**C–G**) Frontal cortex and SN were double immunostained for CYS C (c, g; arrows), VEGF (b, f; arrows) in (**C**), CYS C (c, g; arrows), LC3B (b, f; arrows) in (**D**), CYS C (c, g; arrows), SNCA (b, f; arrows) in (**E**), CYS C (c, g; arrows), Ser129-phosphorylated SNCA (b, f; arrows) in (**F**), CYS C (c, g; arrows), cleaved CASP3 (b, f; arrows) in (**G**), respectively. Double labeling showed that CYS C, VEGF and LC3B immunoreactivity were stronger in cystatin C-treated Tg mice than Tg mice, while SNCA, Ser129-phosphorylated SNCA and cleaved CASP3 immunoreactivity was less in cystatin C-treated Tg mice than non-Tg mice both in the frontal cortex and SN (Scale bar, 50 *μ*m)

We observed 5.8-fold and 5.5-fold increases in CYS C expression, 3.75-fold and 3-fold increases in VEGF and 1.86-fold and 3.2-fold NURR1 among the striata and substantia nigra (SN) of CYS C-treated A53T SNCA mice, respectively, compared to A53T SNCA mice (***P<0.001, CYS C-treated A53T *versus* A53T, *n*=5; Figure 1A and B). However, significant 3.1-fold (striatum) and 6.2-fold (SN) decreases in Ser129-phosphorylated SNCA expression were found in CYS C-treated A53T SNCA mice (***P<0.001, CYS C-treated A53T *versus* A53T, *n*=5; Figure 1A and B) compared to A53T SNCA mice. The expression levels of CYS C, VEGF, NURR1 and Ser129-phosphorylated SNCA in the frontal cortex and hippocampus showed similar trends to those in the striatum and SN (Figure 1A and B).

LC3B and SQSTM1, robust markers of autophagosomes and autophagy substrate, respectively, were used to measure autophagic induction in A53T SNCA mice. Our data showed significant 2.9-fold (frontal cortex), 3.53-fold (striatum), 1.5-fold (hippocampus) and 3.2-fold (SN) decreases in the levels of LC3B-II/LC3B-I expression in CYS C-treated A53T SNCA mice compared to A53T SNCA mice (**P<0.01, ***P<0.001, CYS C-treated A53T *versus* A53T, *n*=5; Figure 1A and B). In contrast, we observed that SQSTM1 decreased by 3.4-fold (frontal cortex), 1.6-fold (striatum), 2.1-fold (hippocampus) and 4.3-fold (SN) in CYS C-treated A53T SNCA mice compared to A53T SNCA mice (***P<0.001, CYS C-treated A53T *versus* A53T, *n*=5; Figure 1A and B).

Immunofluorescence with confocal microscopy in the frontal cortex and SN indicated that CYS C co-localized with VEGF, NURR1 and the autophagsome marker LC3B (Figure 1C–G). Double labeling showed that the immunoreactivity of VEGF, NURR1 and LC3B were stronger in CYS C-treated A53T SNCA mice than in the A53T SNCA mice; while the immunoreactivity of accumulated endogenous SNCA, Ser129-phosphorylated SNCA, the apoptosis marker-cleaved caspase-3 (cleaved CASP3) were lower in CYS C-treated A53T SNCA mice than in the A53T SNCA mice (Figure 1C–G).

### *In vitro* study: CYS C’s essential function in neurovascular interactions

#### CYS C knockdown enhanced apoptosis and inflammation in 6-OHDA-lesioned DAergic PC12 cells

**Figure 2:**
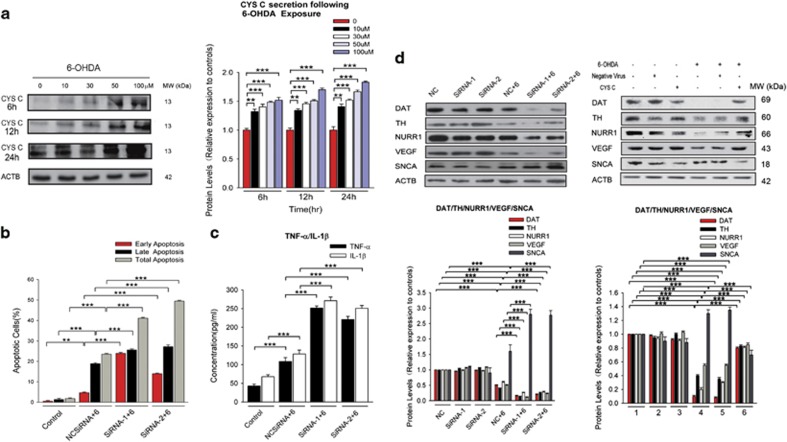
Knockdown of ***Cst3*** promotes apoptosis and neurodegeneration, while overexpression of CYS C has a neuroprotective effect in an *in vitro* PD model. (**a**) 6-OHDA induces a time-dependent and concentration-dependent change in the expression of CYS C in PC12 cells. Western blot analysis shows CYS C levels in PC12 cells after treatment with 6-OHDA at different concentrations (0, 10, 30, 50, 100 *μ*M) for various times (0, 6, 12, 24 h). The diagram shows the relative quantitation of CYS C protein levels compared with that of ACTB. The data of each time group are expressed as the relative ratios of the 0 *μ*M group, which were set to 1.0, and are expressed as the mean±S.E.M. of five independent experiments. **P<0.01, ***P<0.001. (**b–d**) For blocking or enhancing CYS C function, *Cst3* siRNA oligonucleotides and lentiviral vectors carrying CYS C were added to 6-OHDA-incubated PC12 cells, respectively. (**b**) Knockdown of *Cst3* promotes cellular apoptosis. The bar chart shows the apoptotic rate of PC12 cells (Supplementary Fig. 1). The results are expressed as the mean±S.E.M. of five independent experiments. **P<0.01, ***P<0.001. (**c**) Knockdown of *Cst3* increases the level of inflammatory-related mediators. The bar chart shows the concentration of inflammatory cytokine levels of TNF-*α* and IL-1*β*. ***P<0.001. (**d**) Knockdown of *Cst3* decreases the protein levels of TH, DAT, VEGF and NURR1, as well as a significant increase in SNCA expression measured by western blot, while overexpression of CYS C reversed such phenomenon. The bar chart shows the relative quantification of the specific protein levels compared with that of ACTB. The data are expressed as the relative ratios of the blank group, which was set to 1.0, and are expressed as the mean±S.E.M. of five independent experiments. ***P<0.001

CYS C levels were increased in a time and dose-dependent manner in 6-OHDA-lesioned PC12 cells, with a 24 h 6-OHDA (100 *μ*M) incubation producing the most obviously increase in CYS C release compared to the other groups (Figure 2a). Therefore, we chose to use this concentration in the subsequent experiments. The total apoptotic values (Figures 2b and 4c,Supplementary Figure 1) and the expression of TNF-*α* and IL-1*β* were increased (Figure 2c), while the expression levels of the DAergic PC12 cells markers DAT and TH were significantly reduced (Figure 2d), in 6-OHDA-incubated PC12 cells with *Cst3* knockdown, compared to those cells incubated with only 6-OHDA. These data suggest that CYS C knockdown aggravates apoptosis and inflammation in DAergic PC12 cells.

#### Overexpression of CYS C attenuated PC12 cell degeneration by regulating VEGF-p-PKC-*α*/p-ERK1/2-NURR1 signaling

**Figure 3:**
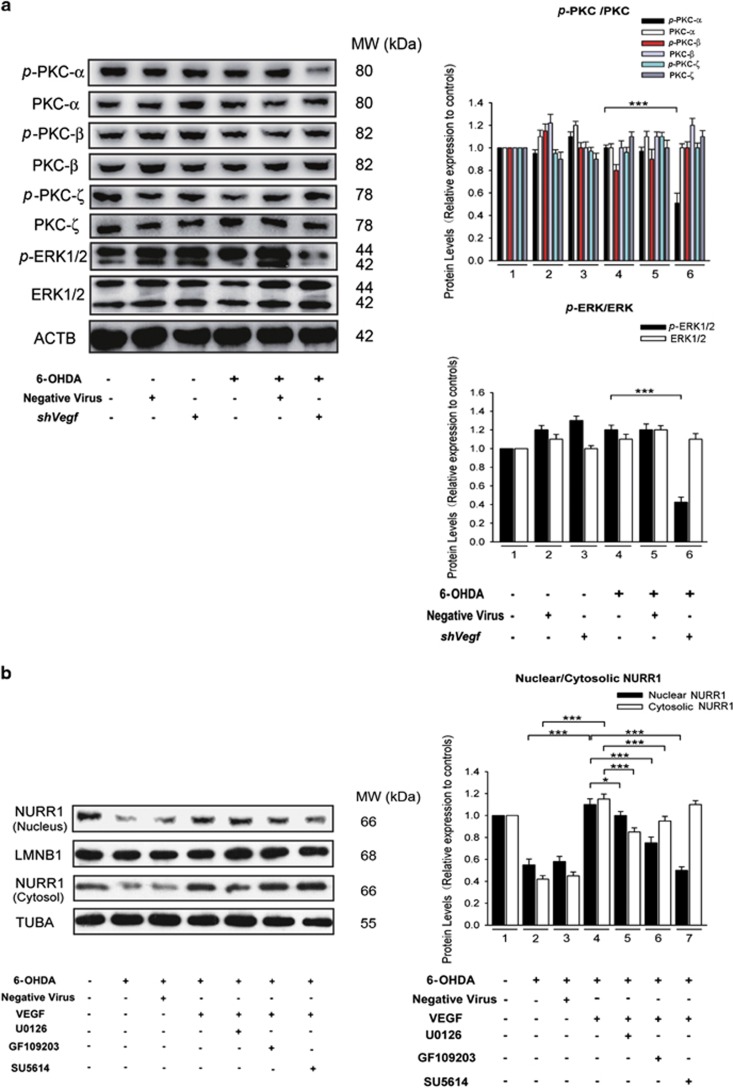
CYS C attenuates neuronal degeneration by regulating VEGF-*p*-PKC-*α*/*p*-ERK1/2-Nurr1 signaling. (**a**) Knockdown of *Vegf* by lentivirus mediated *Vegf* shRNA downregulated the expression of p-PKC-*α* and p-ERK1/2, while it had no effects on the expression of other subtypes of p-PKC (p-PKC-*β*, p-PKC-*ζ*). The bar chart shows the relative quantification of the specific protein levels compared with that of ACTB. The data are expressed as the relative ratios of the blank group, which was set to 1.0, and are expressed as the mean±S.E.M. of five independent experiments. ***P<0.001. (**b**) PC12 cells were nontransfected or transfected with negative virus or lentiviral vector for 3–4 d and then were incubated with 100 *μ*M 6-OHDA for another 24 h, with or without the inhibitors (U0126, GF109203 and SU5614) for ERK, PKC and VEGFR2/KDR, respectively. The lysates from the cytosol and nucleus were probed for NURR1 and normalized to TUBA (cytosolic marker; internal control) and LMNB1 (nuclear marker; internal control). Overexpression of VEGF led to upregulation of both nuclear and cytosolic NURR1 protein expression. VEGFR2/KDR contributed most to VEGF-regulated nuclear NURR1 gene expression, followed by the PKC pathway, and the ERK pathway contributed the least. When the VEGF-regulated nuclear NURR1 pathway was blocked, NURR1 translocated to the cytosol from the nucleus, leading to an opposite trend in cytosolic NURR1 protein expression compared with the nuclear NURR1 protein expression. The bar chart shows the relative quantification of the specific protein levels compared with that of ACTB. The data are expressed as the relative ratios of the blank group, which was set to 1.0, and are expressed as the mean±S.E.M. of five independent experiments. *P<0.05, ***P<0.001

NURR1 is an orphan nuclear receptor that has been characterized as a transcription factor important for DAergic neuron development.^19^ Recent studies have shown that VEGF potently induces the expression of NURR1 in endothelial cells.^20^ Interestingly, we found overexpression of CYS C increases NURR1 and VEGF expression (***P<0.001, *n*=5; Figure 2d). In addition, 6-OHDA incubation significantly decreased the density of cytosolic and nuclear NURR1 protein, while VEGF overexpression obviously restored the density of cytosolic and nuclear NURR1 to normal control levels (*P<0.05, ***P<0.001, *n*=5; Figure 3b). These results indicate that VEGF may act as a mediator between CYS C and downstream NURR1 expression. Previous reports also showed that Nurr1 can be phosphorylated by the ERK1/2 and PKC signaling pathways and translocate to the nucleus, where it is activated.^21, 22, 23^ Our observations led us to explore whether VEGF exerts its effects on NURR1 by regulating ERK and PKC signaling. Following 6-OHDA incubation, knockdown of *Vegf* led to significant (50% and 62%, respectively) decreases in the expression of p-PKC-*α* and p-ERK1/2, as compared to 6-OHDA incubation alone (***P<0.001, *n*=5; Figure 3a). Following 6-OHDA incubation, U0126 (an inhibitor of ERK1/2), GF109203 (an inhibitor of PKC), or SU5614 (an inhibitor of VEGFR2/KDR) incubation significantly attenuated this VEGF overexpression-induced restoration of nuclear NURR1 protein expression, with SU5614 displaying the most pronounced effect (*P<0.05, ***P<0.001, *n*=5; Figure 3b). These data suggested that VEGFR2/KDR contributes the most to VEGF-regulated nuclear NURR1 expression, followed by the PKC pathway. When the VEGF-regulated nuclear NURR1 pathway was blocked, NURR1 translocated to the cytosol from the nucleus, leading to an opposite trend in cytosolic NURR1 protein expression compared with the nuclear NURR1 protein expression. These results indicate that overexpression of CYS C in 6-OHDA-lesioned DAergic PC12 cells profoundly attenuates PC12 cellular degeneration, probably through translocation of NURR1 from the cytosol to the nucleus via VEGF-*p*-PKC-*α*/*p*-ERK1/2 signaling.

#### Overexpression of CYS C in 6-OHDA-lesioned DAergic PC12 cells attenuated PC12 cell degeneration by upregulating VEGF-mediated autophagy

**Figure 4:**
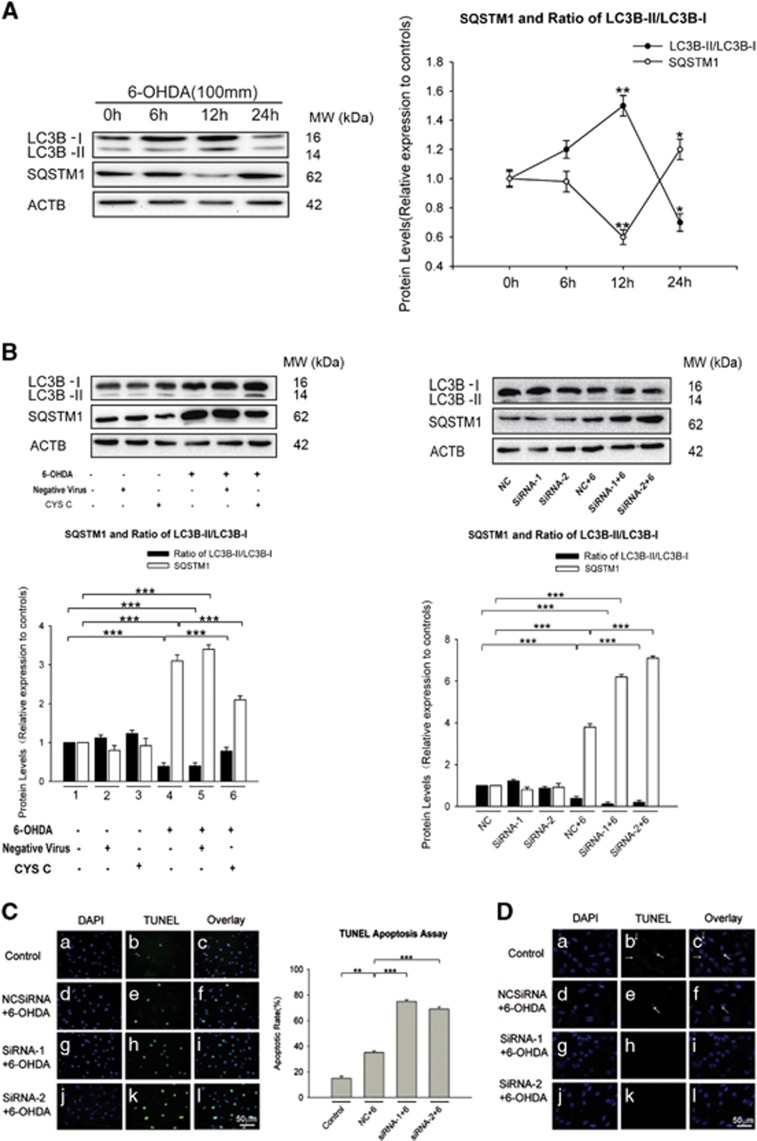

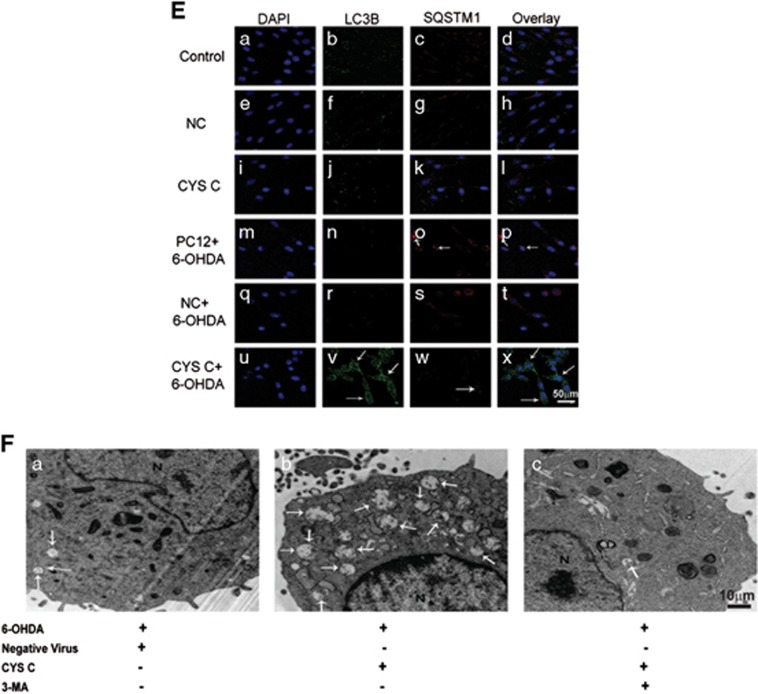
CYS C promotes neuronal survival by regulating neuronal autophagy. (**A**) 6-OHDA induced changes in autophagy signals. The data of each time group are expressed as the relative ratios of the 0 *μ*M group, which were set to 1.0, and are expressed as the mean±S.E.M. of five independent experiments. *P<0.05, **P<0.01, Bonferroni’s *t*-test *versus* 0 h. (**B**) Overexpression of CYS C induced a significant accumulation of LC3B-II and downregulation of SQSTM1; while knockdown of *Cst3* showed an opposite trend. The bar chart shows the relative quantitation of specific protein levels compared with that of ACTB. The data are expressed as the relative ratios of the blank group, which was set to 1.0, and expressed as the mean±S.E.M. of five independent experiments. ***P<0.001. (**C** and **D**) PC12 cells were non-transfected or transfected with NC or siRNAs for 48 h and then were incubated with 100 *μ*M 6-OHDA for another 24 h, followed by TUNEL (terminal deoxynucleotidyl transferase dUTP nick-end labeling) staining to detect apoptotic cells (green, c), and immunostaine for LC3B to detect autophagy (red, **D**). The ratio of TUNEL-positive cells in PC12 cells was calculated. **P<0.01, ***P<0.001 (Scale bar: 50 *μ*m). (**E**) PC12 cells were double immunostained for LC3B and SQSTM1. A stronger LC3B immunoreactivity and less SQSTM1 immunoreactivity were found in 6-OHDA-lesioned PC12 cells with CYS C-overexpression compared with 6-OHDA-lesioned PC12 cells (Scale bar: 50 *μ*m). (**F**) Introduction of overexpression of CYS C increased the number of autophagic vesicles. Arrows indicate autophagic vesicles. N, nucleus (Scale bar: 10 *μ*m)

We observed that 6-OHDA induced early autophagy (starting at 6 h) through LC3B-II conversion and a significant increase in the LC3B-II conversion rate was present at 12 h; however, 6-OHDA then inhibited autophagy at 24 h of incubation (Figure 4A). These results suggest that 6-OHDA treatment causes a biphasic change in autophagy: it initially leads to autophagic induction, followed by decreased autophagy with 24 h of incubation (*P<0.05, **P<0.01, *n*=5; Figure 4A). This finding was consistent with the study by In *et al.*^24^

To test whether CYS C regulated autophagy in 6-OHDA-lesioned DAergic PC12 cells, we overexpressed CYS C in 6-OHDA-lesioned PC12 cells. CYS C overexpression significantly unregulated the levels of LC3B-II/LC3B-I by 1.75-fold and downregulated the levels of SQSTM1 by 1.8-fold compared to cells without CYS C overexpression (***P<0.001, *n*=5; Figure 4B), while *Cst3* knockdown yielded the opposite trend in LC3B-II and SQSTM1 expression (***P<0.001, *n*=5, Figure 4B). Our immunofluorescence findings showed results consistent with these findings for the expression levels of LC3B and SQSTM1, as indicated in Figure 4D and E. Furthermore, our transmission electron microscopy (TEM) analysis demonstrated that overexpressing CYS C in 6-OHDA-lesioned PC12 cells increased the number of autophagic vesicles (Figure 4Fa and c). Taken together, our data strongly indicates that overexpression of CYS C upregulated autophagy in the 6-OHDA-lesioned PC12 model.

**Figure 5:**
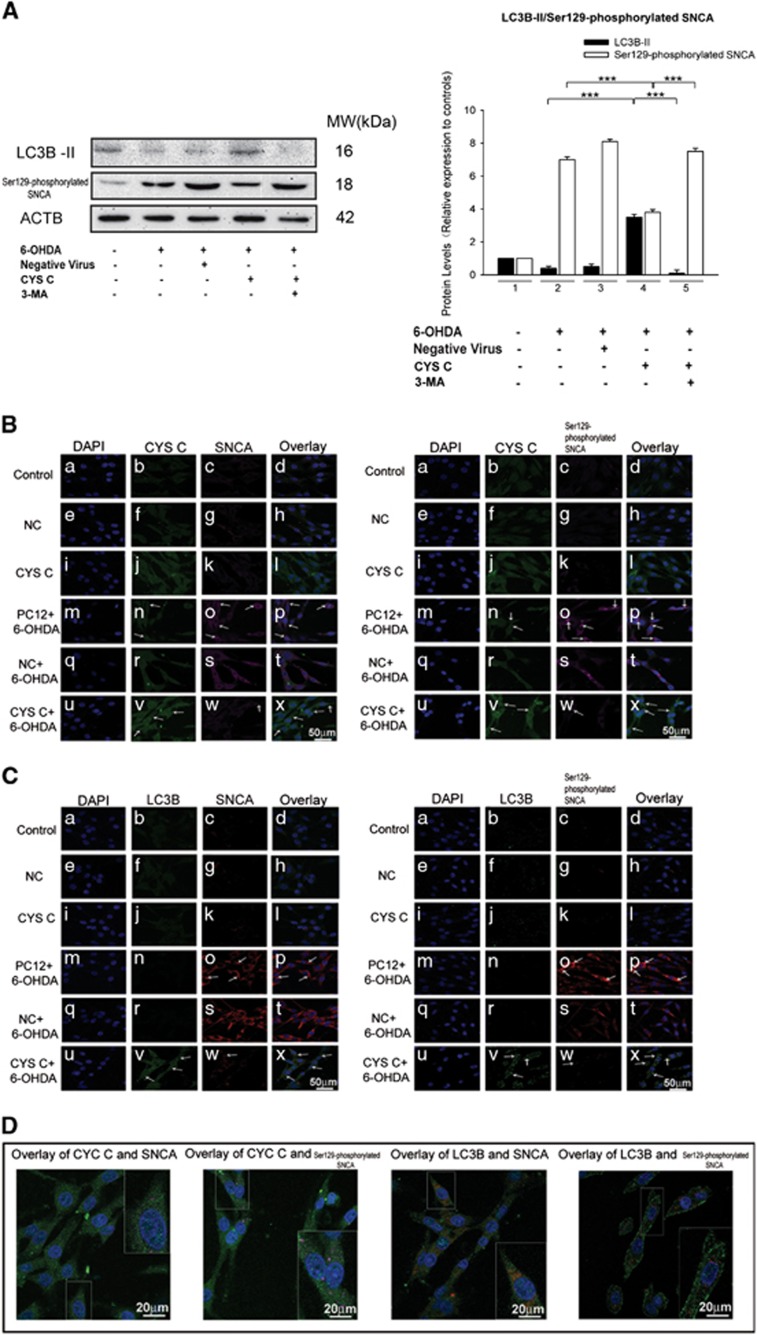

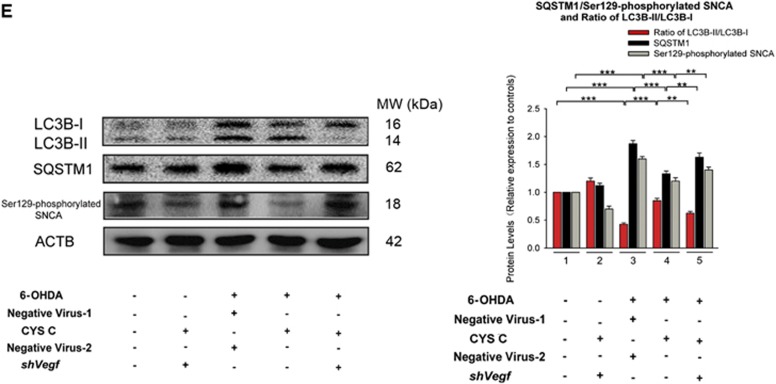
CYS C promotes neuronal survival partially through VEGF-mediated autophagic clearance of SNCA aggregation. (**A**) Inhibition of autophagy with 3-MA induced a significant accumulation of Ser129-phosphorylated SNCA by downregulating LC3B-II. The bar chart shows the relative quantitation of specific protein levels compared with that of ACTB. The data are expressed as the relative ratios of the blank group, which was set to 1.0, and expressed as the mean±S.E.M. of five independent experiments. ***P<0.001. (**B** and **C**) PC12 cells were double immunostained for CYS C and SNCA/Ser129-phosphorylated SNCA in (**B**), LC3B and SNCA/Ser129-phosphorylated SNCA in (**C**), respectively. A stronger CYS C, LC3B immunoreactivity and less SNCA/Ser129-phosphorylated SNCA accumulation were found in 6-OHDA-lesioned PC12 cells with CYS C-overexpression compared with 6-OHDA-lesioned PC12 cells (Scale bar: 50 *μ*m). (**D**) The individual pictures for × (**B**) and × (**C**) are shown. Higher-magnification images of the framed areas are located in the upper/lower right corner (Scale bar: 20 *μ*m). (**E**) *Vegf* knockdown attenuated the effects of CYS C overexpression on SQSTM1, LC3B-II/LC3B-I and Ser129-phosphorylated SNCA degradation under oxidative stress. For enhancing CYS C expression, lentiviral vectors carrying *Cst3* and Negative Virus-1 were added to 6-OHDA-incubated PC12 cells, respectively. Knockdown of *Vegf* was mediated by lentivirus carrying *Vegf* shRNA and Negative Virus-2. The bar chart shows the relative quantitation of specific protein levels compared with those of ACTB. The data are expressed as the relative ratios of the blank group, which was set to 1.0, and expressed as the mean±S.E.M. of five independent experiments. **P<0.01, ***P<0.001

Recent studies have revealed an enrichment of Ser129-phosphorylated SNCA is found in Lewy bodies, suggesting that Ser129 phosphorylation is involved in the pathogenesis of PD.^25^ In addition, Ser129-phosphorylated SNCA/SNCA is degraded mainly by the autophagy pathway.^26, 27^ In accordance with these findings, our western blot data showed that CYS C overexpression significantly downregulated SNCA*/*Ser129-phosphorylated SNCA levels (***P<0.001, *n*=5; Figures 2d and 5A); while 3-MA treatment (an autophagy inhibitor) significantly reduced the number of autophagic vesicles and attenuated the CYS C overexpression-induced downregulation of SNCA*/*Ser129-phosphorylated SNCA levels (***P<0.001, *n*=5; Figures 4F and 5A). Strikingly, we noted that the immunoreactivity of the autophagy marker LC3B was discordant with that of SNCA*/*Ser129-phosphorylated SNCA levels under oxidative stress conditions and the opposite pattern was observed with CYS C overexpression (Figure 5B,C(n, o, v, w) and D). Therefore, we reasonably concluded that CYS C promotes 6-OHDA-lesioned DAergic PC12 cell survival by enhancing autophagic clearance of SNCA aggregates.

The potential association among microvascular endothelial cells, VEGF and autophagy has been previously documented.^28, 29^ To assess the effects of VEGF on CYS C overexpression-mediated enhancement of autophagy, we knocked down *Vegf* by lentivirus-mediated *Vegf* short hairpin ribonucleic acid (shRNA) after overexpression of CYS C in PC12 cells under oxidative stress conditions. Interestingly, we observed that the levels of LC3B-II/LC3B-I in 6-OHDA-incubated PC12 cells with *Vegf* knockdown after overexpression of CYS C was significantly reduced by 33%, compared to those with only CYS C overexpression (**P<0.01, ***P<0.001, *n*=5; Figure 5E). The expressions of SQSTM1 and Ser129-phosphorylated SNCA were also significantly increased by 1.2-fold and 1.16-fold, respectively (**P<0.01, ***P<0.001, *n*=5; Figure 5e). Our data demonstrates that VEGF mediates CYS C-induced autophagic clearance of SNCA/Ser129-phosphorylated SNCA aggregates in 6-OHDA-lesioned PC12 cells.

#### Conditioned media of 6-OHDA-lesioned, CYS C-overexpressing DAergic PC12 cells induced VEGF-mediated angiogenesis

**Figure 6:**
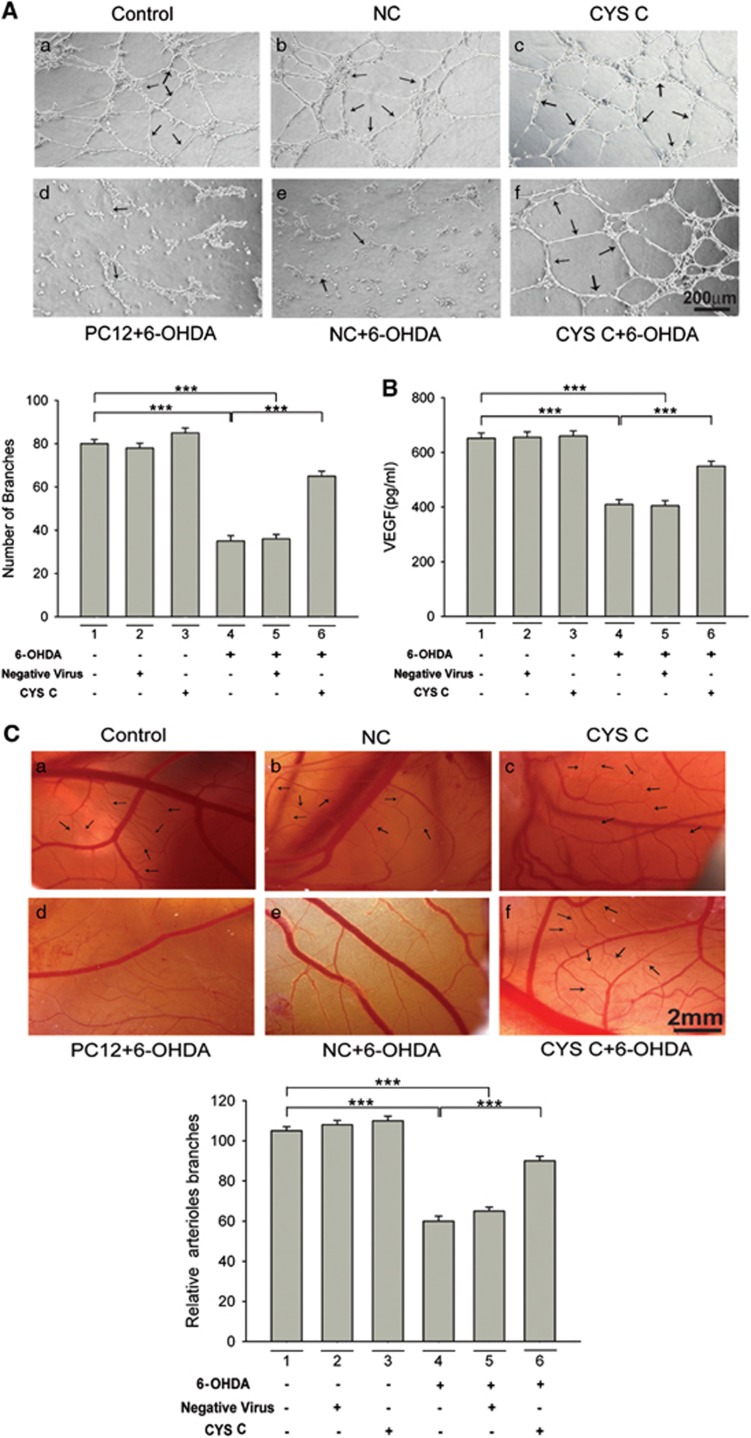

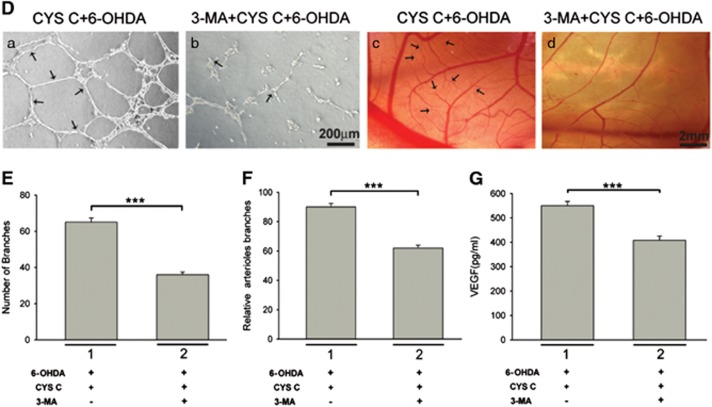
CYS C promotes angiogenesis via regulating VEGF, while CYS C-mediated enhanced autophagy influences VEGF-induced angiogenesis in NVUs. (**A**) Sample images showing the promotional effect of CYS C on HUVEC tube formation in the conditioned media of 6-OHDA-lesioned PC12 cells with CYS C-overexpression (Scale bar: 200 *μ*m). The bar chart shows the number of branch points of HUVECs. The results are expressed as the mean±S.E.M. of five independent experiments. ***P<0.001. (**B**) The bar chart shows the concentration of VEGF in the conditioned media of 6-OHDA-lesioned PC12 cells with CYS C-overexpression. The results are expressed as the mean±S.E.M. of five independent experiments. ***P<0.001. (**C**) Fertilized eggs were treated with PC12 cell-conditional media from (a) PC12 cells without transfection, (b) PC12 cells transfected with NC, (c) PC12 cells transfected with overexpressing CYS C, (d) 6-OHDA-lesioned PC12 cells (*in vitro* model of PD) without transfection, (e) 6-OHDA-lesioned PC12 cells transfected with NC and (f) 6-OHDA-lesioned PC12 cells transfected with overexpressing CYS C (Scale bar: 2 mm). Angiogenesis was quantified by counting the number of arteriole branches in the bar chart. The data are presented as the mean±S.E.M., based on three independent experiments. ***P<0.001. (**D–G**) Blockage of autophagy in 6-OHDA-lesioned PC12 cells with CYS C-overexpression in turn downregulated the level of VEGF and attenuated VEGF-mediated angiogenesis. (**D**) The TF assay (a, b) and CAM assay (c, d) both showed the conditioned media from CYS C-overexpressing PC12 cells incubated with 3-MA and 6-OHDA significantly inhibited the formation of branched blood vessels. (Scale bar: 200 *μ*m; 2 mm). The bar chart shows the number of branch points of HUVECs in (**E**), the number of arteriole branches in (**F**) and the concentration of VEGF in (**G**), respectively. The results are expressed as the mean±S.E.M. of five independent experiments. ***P<0.001

In the current study, we found that VEGF may be a downstream mediator of CYS C in 6-OHDA-lesioned PC12 cells. The results of an ELISA (Figure 6B) showed that VEGF levels dramatically increased in the conditioned media of 6-OHDA-lesioned, CYS C-overexpressing PC12 cells. We further identified the effect of this conditioned media on angiogenesis *in vitro* (Figure 6A). When human umbilical vein endothelial cells (HUVECs) were placed on Matrigel, robust and elongated tube-like structures were formed after incubation in the conditioned media from 6-OHDA-lesioned PC12 cells with CYS C-overexpression (Figure 6Af).

We next sought to examine whether **CYS C** could also promote angiogenesis *in vivo*. As shown in Figure 6C, angiogenesis was clearly observed in fertilized eggs after a 24 h of treatment with the conditioned media, and the group treated with the conditioned media from 6-OHDA-lesioned PC12 cells with CYS C-overexpression significantly promoted the formation of branched blood vessels compared to the untreated Chick Embryo Chorioallantoic Membrane (CAMs) (Figure 6Cf). Based on these results, we conclude that, in both *in vitro* and *in vivo* systems, VEGF expression and VEGF-mediated angiogenesis markedly increased upon exposure to conditioned media of 6-OHDA-lesioned, CYS-C overexpressing PC12 cells.

#### Blockage of autophagy in CYS C-overexpressing DAergic PC12 cells treated with 6-OHDA reduced VEGF-induced angiogenesis

To further confirm CYS C’s essential function in neuro-vascular interactions *in vitro*, we blocked autophagy in CYS C-overexpressing PC12 cells and examined whether CYS C overexpression attenuated angiogenesis via downregulating the levels of secreted VEGF. As shown in Figure 6D, the tube formation rate in the group treated with the conditioned media of CYS C-overexpressing PC12 cells incubated with 3-MA and 6-OHDA was decreased to 56±10.1% of the group without 3-MA treatment (***P<0.001; Figure 6D(b and e)); our CAM assay findings also showed that the conditioned media from CYS C-overexpressing PC12 cells incubated with 3-MA and 6-OHDA significantly inhibited the formation of branched blood vessels *in vivo* (***P<0.001; Figure 6D(d and f)). The ELISA results (Figure 6G) demonstrated that VEGF levels dramatically decreased in the conditioned media with 3-MA treatment. These data suggested that blockage of autophagy in 6-OHDA-lesioned PC12 cells with CYS C-overexpression in turn reduced the VEGF expression and subsequently downregulated VEGF-induced angiogenesis both *in vitro* and *in vivo*. Taken together, we confirmed that CYS C-induced autophagy in DAergic PC12 cells had neuronal-vascular dual functions, promoting PC12 cell survival and angiogenesis via regulating the level of secreted VEGF protein.

## Discussion

CYS C has been previously documented to play important roles in the pathogenesis of AD, vascular dementia (VaD) and ALS.^17, 30, 31^ However, the exact mechanism still remains unclear and needs extensive studies to explore. The current study shows that CYS C is a potential mediator functioning to induce angiogenesis and enhance cellular autophagy in the NVUs of PD models. We obtained four principal findings in this study: (1) we observed increased VEGF, NURR1 and autophagy markers LC3B and decreased SNCA and apoptosis marker cleaved CASP3 in different brain regions of CYS C-treated A53T SNCA transgenic mice; (2) in an *in vitro* study, we confirmed CYS C’s pivotal functions in the NVUs. In detail, CYS C overexpression upregulated the levels of VEGF, while CYS C-induced VEGF attenuated 6-OHDA-lesioned PC12 cell degeneration by regulating p-PKC-*α*/p-ERK1/2-Nurr1 signaling and inducing enhanced autophagy; (3) in the NVUs, as a secreted protein, VEGF in the conditioned media of 6-OHDA-lesioned PC12 cells overexpressing CYS C markedly increased angiogenesis. Interestingly, blockage of autophagy by 3-MA in the CYS C-overexpressing PC12 cells significantly decreased VEGF expression and VEGF-mediated angiogenesis. Taken together, we propose that CYS C has neuronal–vascular dual functions, promoting PC12 cell survival and angiogenesis, via regulating the levels of secreted VEGF protein in the NVUs.

A53T SNCA transgenic mice were used here as an *in vivo* PD model.^32, 33^ Interestingly, the upregulated expression of VEGF, NURR1 and autophagy markers LC3B, as well as decreased Ser129-phosphorylated SNCA and apoptosis marker cleaved CASP3 were observed in CYS C-treated A53T SNCA transgenic mice. These findings strongly imply that CYS C is involved in DA neuroprotection as indicated by the upregulation of VEGF, NURR1 and downregulation of Ser129-phosphorylated SNCA following CYS C injection into the SN of A53T SNCA transgenic mice; while CYS C-mediated neuroprotection is also associated with enhanced autophagy, as shown by the upregulated LC3B and downregulated SQSTM1 in the CYS C-treated A53T SNCA transgenic mice. Moreover, we also observed that CYS C co-localized with VEGF, NURR1 and autophagy markers in the CYS C-treated A53T SNCA transgenic mouse brains, including the frontal cortex and SN (Figure 1A–G). These findings provide a hypothesis that CYS C may participate in NVU activity via interacting with VEGF and autophagy pathways.

Based on our murine studies, we further investigated CYS C’s functions in the NVUs *in vitro*. We found the overexpression of CYS C increases VEGF expression and VEGF overexpression significantly restored the 6-OHDA-mediated downregulation of both nuclear and cytosolic Nurr1 proteins, strongly indicating that VEGF may act as a mediator between CYS C and downstream NURR1 expression and that the upregulation of VEGF by CYS C overexpression might promote DAergic neuronal survival. It has been well documented that ERK and PKC signaling pathways are associated with neuronal survival, and previous studies have suggested that it is correlated with NURR1.^34^ Consistent with these findings, our *in vitro* data verify (Figure 3a and b) that CYS C-induced VEGF attenuated 6-OHDA-lesioned DAergic PC12 cells degeneration by regulating *p*-PKC-*α*/*p*-ERK1/2-Nurr1 signaling and inducing autophagy.

Our observation that 24 h of incubation with 6-OHDA inhibits autophagy in PC12 cells is consistent with In *et al.*’s study.^9^ Recent studies have reported that SNCA is a crucial factor in PD pathogenesis,^33, 35, 36, 37^ and it is usually recognized as a hallmark of PD. Its phosphorylation could accelerate PD neurodegeneration,^38^ and the autophagy process could prevent or reverse its phosphorylation.^39, 40^ The current results revealed that CYS C overexpression profoundly attenuated the 6-OHDA-mediated increase in Ser129-phosphorylated SNCA aggregation, and reversed these contra-directional changes for LC3B-II/LC3B-I and SQSTM1, providing clear evidence of the direct function of CYS C in the autophagic clearance of SNCA aggregation in the *in vitro* PD model. This enhanced autophagy and Ser129-phosphorylated SNCA degradation induced by CYS C overexpression were completely abolished by the autophagy inhibitor 3-MA; similar findings were observed in our TEM data, further verifying the enhancement in autophagy by CYS C overexpression. On the other hand, *Cst3* knockdown in 6-OHDA-lesioned PC12 cells produced the opposite changes in LC3B-II/LC3B-I and SQSTM1 levels compared to those with CYS C overexpression, verifying this relationship. More importantly, we noted that *Vegf* knockdown was able to partially attenuate the effects of CYS C overexpression on enhanced autophagy and Ser129-phosphorylated SNCA degradation under oxidative stress, strongly implying that CYS C-induced VEGF attenuates DAergic PC12 cell degeneration by enhancing autophagic clearance of SNCA aggregates. These findings strongly imply that CYS C promotes neuronal survival partially through VEGF-mediated enhanced autophagy.

As a secreted protein, VEGF can exert its functions on both neural cells and the surrounding cerebral microenvironment, for example, by regulating vascular and neural differentiation, proliferation and survival during development.^9, 10, 11^ To explore the above hypothesis obtained from our *in vivo* studies, we used the tube formation (TF) assay and CAM assay. The conclusion that CYS C regulates angiogenesis is drawn from the observations in the TF assays that the branch points of the capillary-like structures markedly decreased with exposure to PC12 cell-conditioned media incubated with 6-OHDA compared to PC12 cell-conditioned media without 6-OHDA. Furthermore, HUVECs developed more capillary-like structures with exposure to conditioned media of PC12 cells overexpressing CYS C, and it was noted that VEGF expression was markedly increased in this conditioned media. These results demonstrated that CYS C has positive effects on VEGF-mediated angiogenesis *in vitro.* In addition, in the CAM assay, usually recognized as an *in vivo* study, increased branched vessel formation was observed with exposure to conditioned media from 6-OHDA-lesioned PC12 cells overexpressing CYS C compared to those without CYS C overexpression. Consistent with previous studies showing that angiogenesis was pivotal for neuron–vascular survival,^11, 41, 42, 43, 44^ our *in vitro* and i*n vivo* findings indicate that CYS C exerts angiogenesis functions via regulating the level of secreted VEGF protein in the NVUs.

To further confirm CYS C’s function in neuro–vascular interactions, we blocked autophagy in CYS C-overexpressed DAergic PC12 cells and examined whether it attenuates angiogenesis via regulating the level of secreted VEGF. It is noteworthy that in the TF and CAM assay, the overexpression of CYS C in 6-OHDA-lesioned PC12 cells upregulated the level of VEGF and VEGF-induced angiogenesis; while blockage of autophagy in 6-OHDA-lesioned PC12 cells overexpressing CYS C downregulated the level of VEGF and attenuated VEGF-mediated angiogenesis. Our findings are consistent with the study by Poehler *et al.*,^12^ showing that enhanced autophagy not only regulates secreted proteins but also ameliorates the micro-environmental responses to cellular damage. Taken together, we reasonably propose that CYS C-induced autophagy in DAergic PC12 cells display neuronal–vascular dual functions of promoting PC12 cell survival and inducing angiogenesis via regulating the secreted VEGF protein in the NVUs. As we know that NUVs consist of multiple cell types such as neurons, endothelial cells, astrocytes and microglia.^1, 2^ In this study, we mainly focused on neurons and endothelial cells. It is worthy of conducting further in-depth studies in the future to explore the effects of CYS C on other cell types in NUVs, that is, astrocytes and microglia.

**Figure 7:**
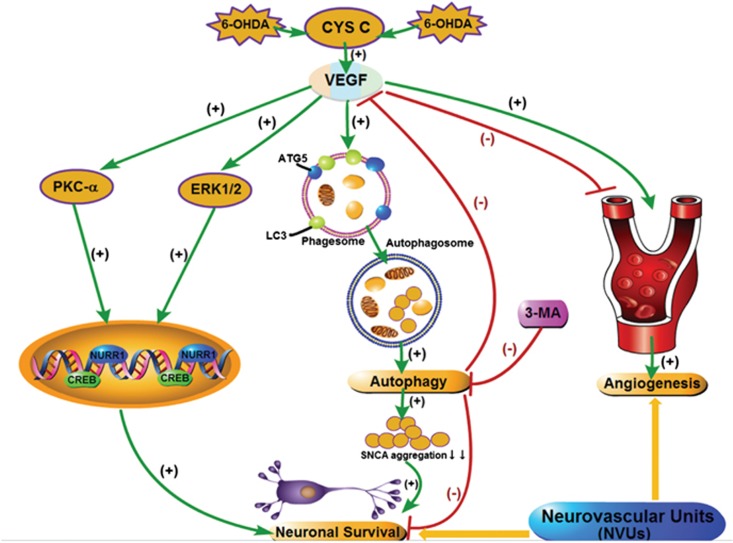
Schematic shows the signaling mechanisms underlying the –vascular dual functions of CYS C. CYS C exerts neuronal–vascular dual functions in promoting neuronal survival and angiogenesis via regulating the secreted protein VEGF in NVUs. However, blockage of autophagy in CYS C-overexpressing DAergic PC12 cells significantly aggravates neuronal degeneration by increasing SNCA aggregation and attenuates VEGF-mediated angiogenesis in NVUs

In conclusion, our study demonstrates that CYS C displays a neuroprotective effect in the A53T SNCA transgenic mice by upregulating VEGF and autophagy and downregulating a-synuclein and apoptosis. As shown in Figure 7, CYS C-induced VEGF expression attenuated 6-OHDA-lesioned DAergic PC12 cell degeneration by regulating *p*-PKC-*α*/*p*-ERK1/2-NURR1 signaling and inducing autophagy (Figure 7). VEGF-mediated angiogenesis was markedly enhanced upon exposure to conditioned media from 6-OHDA-lesioned PC12 cells overexpressing CYS C. The blockage of autophagy in CYS C-overexpressing DAergic PC12 cells significantly downregulated secreted VEGF expression and subsequently attenuated VEGF-mediated angiogenesis, strongly indicating that CYS C-mediated enhanced neuronal autophagy plays an important role in the NVUs. Importantly, we propose that CYS C has the neuronal–vascular dual functions of promoting PC12 cells survival and angiogenesis via regulating the level of secreted VEGF protein in the NVUs. These *in vitro* and *in vivo* findings suggest that CYS C could be used as a novel angiogenesis target in clinical applications in PD. In addition, our findings further confirm our hypothesis that CYS C participates in NVU activity via interacting with the VEGF and autophagy pathways. This study provides a clue for the development of an alternative approach to the treatment of PD through neuronal-vascular protection mediated by CYS C.

## Materials and Methods

### Investigation 1: how CYS C/VEGF levels and autophagy change in A53T SNCA mouse brain tissues

#### Western blot analysis and immunofluorescence staining in A53T SNCA mice and CYS C-treated A53T SNCA mice

Transgenic mice expressing the mutant human A53T SNCA under the control of a prion promoter (Prnp-SNCA*A53T),^45^ usually used as a transgenic PD mouse model, were obtained from the State Key Laboratory of Medical Genetics of Central South University (Changsha, China), and the wild-type littermates were used as the controls. We certify that the mice in our study were carried out in accordance with the National Institute of Health Guide for the Care and Use of Laboratory Animals (NIH Publications No. 80–23) revised 1996 guidelines. The protocol was approved by the Institutional Animal Care and Use Committee (Animal Ethic Approval No: 0014102402). We further attest that all efforts were made to minimize the number of animals used and their suffering. The genotypes of all of the wild-type and A53T SNCA transgenic mice were determined by polymerase chain reaction (PCR) amplification analysis using tail DNA at three weeks of age and were verified at the end of the experiment.^46, 47^

All surgery was performed under Equithesin anesthesia (0.3 ml/100 g) and adequate measures were taken to minimize pain or discomfort. The administration of 5 *μ*g human cystatin C (Sigma, St. Louis, MO, USA) in 0.1% bovine serum albumin (BSA) containing phosphate-buffered saline (PBS) or saline containing 0.1% BSA as vehicle was performed. The animals was anesthetized and placed in a stereotaxic frame (ASI Instrument, Warren, MI, USA) as described previously, and all injections were using a 10-*μ*l syringe at a rate of 0.4 *μ*l/min. The injections of vehicle and cystatin C were administered directly into the right SN using the following coordinates: AP, +0.9 mm; *L*, ±2.0 mm; and *V*, −3.0 mm from skull.^48, 49, 50^ At the completion of each injection, the needle was left in place for 5 min and then withdrawn at a rate of 1 mm/min. Four weeks after surgery, western blot and immunofluorescence staining were performed according to the previously published protocols.^51, 52^ Frontal cortex, striatum, hippocampus and SN were selected. For additional details refer to the Supplementary Information.

### Investigation 2: whether CYS C exerts neuronal–vascular dual functions by influencing VEGF-mediated angiogenesis and autophagy in NVUs *in vitro*

#### Cell culture and treatments

The 6-OHDA-lesioned DAergic PC12 cells have been widely used as an *in vitro* PD model, since PC 12 cells could mimic the pathological and biochemical characteristics of PD *in vitro* condition.^53, 54, 55, 56^ They can be used to define important cellular actors of cell death presumably critical for the DAnergic degeneration. The PC12 cells were seeded in 96-well plates or 6-well plates at a density of 1.0 × 10^5^ cells/ml for 24 h. PC12 cells were subjected to different concentrations of 6-OHDA (0, 10, 30, 50, 100 *μ*M) for various time points (0, 6, 12, 24 h). The released CYS C in the PC12 cells was analyzed by western blot. For the measurement of the inflammatory mediators TNF-*α* and IL-1*β*, an enzyme linked immunosorbent assay (ELISA, R&D Systems Inc., Minneapolis, MN, USA) was performed at an absorbance of 570 nm with an ELISA plate reader. Each treatment group was replicated in three wells. All of the results were normalized to optical density (OD) values measured from an identically conditioned well without cells. For enhancing or blocking *Cst3* function, lentiviral vectors carrying *Cst3* and *Cst3* siRNA oligonucleotides were added to 6-OHDA-incubated PC12 cells. In contrast, for enhancing or blocking *Vegf* function, lentiviral vectors carrying *Vegf* and lentivirus mediated *Vegf* shRNA (*shVegf*) were added to 6-OHDA-incubated PC12 cells, respectively.

### Lentiviral vector construction and infection for the overexpression of CYS C and VEGF and shRNA interference for the knockdown of *Vegf*

Lentiviral vectors were used for the overexpression of CYS C and VEGF as previously described with some modifications.^57^ *Cst3*, *Vegf* and green fluorescence protein (GFP) cDNAs were cloned into the pRRL-cPPt-PGK-PreSIN vector. Lentivirus mediated *shVegf* was cloned into p156RRL-sinPPT- CMV-GFP-PRE/NheI. The shRNA design and sequences are available in the online data supplement. Viruses were produced as described.^58^ PC12 cells were transduced for 24 h with recombinant lentivirus at multiplicities of infection (MOIs) of 50 (overexpression of CYS C), 50 (overexpression of VEGF) and 100 (*shVegf*), in the presence of 10 *μ*g/mL Polybrene. After transduction, the cells were cultured in suspension for 72 h. Overexpression of CYS C or VEGF was verified by flow cytometric analyses (GFP) and immunofluorescence. Knockdown and transduction efficiency in the *Vegf* (*shVegf*) constructs were verified by flow cytometric analysis (GFP) and were confirmed by RT-PCR and immunofluorescence.

### Construction of the Cst3 siRNA sequence and its transfection into PC12 cells

Two *Cst3* siRNA oligonucleotides were purchased and identified using the primers S1 (5′-CCATACAGGTGGTGAGAGCTCdTdT-3′) and S2 (5′-GTACCAAGTCCCAGACAAATTdTdT-3′). The negative control sequence (Sn, UUCUCCGAAC GUGUCACGUUUGUGC) was formulated and synthesized. Each sequence (100 nM) was transfected into the PC12 cell line (1 × 10^5^ cells/ml) using the oligofectamine liposome. The cells were divided into four groups: blank control, negative control, S1 transfection (S1) and S2 transfection (S2). There were no differences in the treatments of each group, with the exception that the blank control and negative control were transfected with PBS and empty vector, respectively, at the same working concentrations and volumes. Only the most effective siRNA was used in the subsequent studies.

### Protein extraction, subcellular fractionation and western blot analysis of 6-OHDA-lesioned PC12 cells

The cells were harvested using cell scrapers, washed in ice-cold PBS, and lysed with two different ice-cold lysis buffers. The supernatants were collected for protein determination by BCA assay (Pierce, Inc., Rockford, IL, USA), and the proteins were run on NuPage Bis-Tris 10% gels (Invitrogen, Waltham, MA, USA) and transferred to PVDF membranes (Amersham Bioscience, Ltd., Buckinghamshire, UK). The membranes were blocked in 5% skim milk, 0.05% Tween 20, and Tris-buffered saline (TBS) for 1 h. The PVDF membranes were incubated in the primary antibodies overnight at 4 °C. For detailed antibody information, please refer to the Supplementary Information. The next day, horseradish peroxidase-conjugated secondary antibodies (Cell Signaling, Danvers, MA, USA) were applied. Peroxidase-conjugated streptavidin and substrate were used for detection. Negative controls were prepared by omitting the primary antibodies. For the protein extractions prepared from the cytosolic and nuclear fractions, the method described by Garcia-Yagüe *et al.*^59^ was used. The images were analyzed using NIH Image J software (Bethesda, MD, USA). For additional details, please refer to the Supplementary Information.

### Immunofluorescence in 6-OHDA-lesioned PC12 cells

For immunofluorescence analysis, previously describe methods were employed,^60, 61, 62^ with some modifications. Briefly, 1 × 10^5^ cells/ml from the different experimental groups were plated on confocal Petri dishes in serum-containing media for 24 h. The cells were then incubated in conditioned media alone, 6-OHDA (100 *μ*M) alone, 6-OHDA (100 *μ*M)+*Cst3* knockdown or overexpression of CYS C and 6-OHDA (100 *μ*M)+*Vegf* knockdown or overexpression of VEGF, before staining for immunofluorescence. For additional details, please refer to the Supplementary Information.

### *In vitro* TF assay

The procedure for the *in vitro* capillary-like TF assay was performed as presented in the study by Fang *et al.*,^63^ with some modifications. Briefly, Matrigel (356231, BD Bioscience, San Jose, CA, USA) was used to coat culture plates according to the manufacturer’s instructions. Thawed Matrigel at a volume of 150 ml was applied to each well of 24-multiwell plates and was polymerized at 37 °C for 1 h. HUVECs were cultured in the presence of 90% PC12 cell-conditioned media from (1) PC12 cells without transfection; (2) PC12 cells transfected with NC; (3) PC12 cells overexpressing CYS C; (4) 6-OHDA-lesioned PC12 cells without transfection; (5) 6-OHDA-lesioned PC12 cells transfected with NC; (6) 6-OHDA-lesioned PC12 cells overexpressing CYS C; and (7) CYS C-overexpressing PC12 cells incubated with 3-MA and 6-OHDA. Phase contrast images were taken after 12 h. The tube branching was photographed with inverted-phase contrast microscopy, and the number of tube branching points per field was quantified using Image J software. Six fields under × 200 magnification were randomly selected for each well. The results are expressed as the mean±standard deviation of the mean from five independent experiments.

### CAM assay

The procedure for the CAM assay was performed as described in the study by Wang *et al.*,^64^ with modifications. The fertilized chicken eggs were placed in an incubator upon embryogenesis and maintained under constant humidity at 37 °C. On day 8, a square window was opened in the shell after removing 2–3 ml of albumen to detach the CAM from the shell. Substances treated with the compounds being tested were added to the detached CAM that contained PC12 cell-conditioned media from the groups indicated in the experiment for the capillary-like tubular formation assay. The window was sealed with parafilm and incubated for an additional 24 h. After the second incubation, the CAM arteriosus branches in each treatment group were photographed. The angiogenic effect of CYS C overexpression was indicated by the relative numbers of arteriosus branches. The assay was performed five times to ensure reproducibility.

### TEM

TEM was performed for the visualization and quantitation of autophagic vacuoles.^65, 66^ Cells were fixed in 2.5% glutaraldehyde in 0.1 M PBS buffer at 4 °C overnight, and then post-fixed with 1% osmium tetroxide at room temperature for 2 h. After dehydration in a graded series of acetone, the cells were embedded in Epon 812 resin. Ultrathin (60 nm) sections were collected on 200 mesh copper grids, stained with 2% uranyl acetate in 50% methanol for 10 min, followed by 1% lead citrate for 7 min. Subsequently, the sections were stained with uranyl acetate and lead citrate and examined with a transmission electron microscope (Hitachi H-7650, Chiyoda, Tokyo, Japan).

### Statistical analysis

For the *in vitro and in vivo* studies, the data are expressed as the mean±S.E.M. The data related to the human continuous variables, ELISA and flow cytometry analyses, and the different protein quantifications by western blot were analyzed using one-way analysis of variance (ANOVA) followed by Bonferroni’s comparison *post hoc* analysis (SPSS 15.0 program, Chicago, IL, USA). Differences with *P* values of less than 0.05 are regarded as statistically significant.
