# Supplementary material for: Cystatin C as a potential therapeutic mediator against Parkinson’s disease via VEGF-induced angiogenesis and enhanced neuronal autophagy in neurovascular units
Source: Cell Death Dis. 2017 Jun 1;8(6):e2854–. doi: 10.1038/cddis.2017.240 (PMC5520899; doi:10.1038/cddis.2017.240)
Supplement: Supplementary Information [file cddis2017240x1.doc]

**Supplementary data**

**Materials and Methods**

***Western Blot Analysis and Immunofluorescence in the A53T SNCA Mice***

The animals were handled and euthanized in compliance with institutional and national regulations and policies [^1^](#_ENREF_1). Mice enrolled in the study had carefully balanced litters across treatment groups, and the investigators involved in the data gathering and analyses were unaware of genotype and treatment for the duration of the study.

Western blot analysis performed on brain tissues. Briefly, the mice were euthanized by cervical dislocation, and their brains were dissected on ice to harvest the corresponding regions. Brain samples were then homogenized with lysis buffer (PBS with 0.5 mM EDTA, 1% triton, 0.3 M sucrose, 1 mM aprotinin, 1 mM leupeptin, 1 mM benzamidine,10 mM PMSF, 20 mM PNPP, 20 mM b-glycerophosphate disodium salt hydrate, 100 mM sodium fluoride, 100 mM sodium vanadate and 2 mM DTT). The samples were then centrifuged at 14,000 xg at 4°C for 15 min to separate supernatant and pellet. Protein concentrations were determined using a BCA protein assay kit (Pierce, Rockford, IL, USA), and samples were subjected to western blot analysis with anti-bodies as indicated. The protein levels were quantified using Image J software.

For immunofluorescence staining [^2^](#_ENREF_2), the mice were deeply anesthetized with pentobarbital and were perfused with phosphate-buffered saline (PBS) and then with 4% paraformaldehyde in PBS. Their brains were then harvested and post-fixed overnight in 4% paraformaldehyde in PBS. The brains were subsequently cryoprotected by incubation in 30% of sucrose for overnight, and coronal sections 20 μm in size were cut at using a cryostat (Leica, Wetzlar, Germany). For staining, the sections were first quenched for 10 min in 3% H2O2 and 10% methanol and then were washed with PBS and blocked with 3% BSA for 60 min. The sections were then incubated with anti-bodies as indicated overnight at 4°C. After overnight incubation, the sections were washed and incubated with fluorescent-labeled secondary antibodies (Alexa 488-, Alexa 568-, Alexa 555- or Alexa 647-labeled IgG; Invitrogen) for 1 h. After rinsing with PBS, DAPI (300 nmol/L, Invitrogen, Carlsbad, CA, USA) was used for counterstaining for 3-5 min.Stained sections were then mounted and dried on gelatinized slides overnight and were dehydrated and coverslipped. Confocal analysis was performed using a Zeiss LSM 710/Meta Station (Carl Zeiss) equipped with a digital camera (Hamamatsu, Japan) and operated with QED imaging software.

***Cell Culture and Treatments***

Briefly, PC12 cells were routinely maintained in Dulbecco's modified Eagle’s medium (DMEM) supplemented with 10% fetal bovine serum, 5% horse serum, 100 U/mL benzyl penicillin, and 100 mg/L streptomycin (Gibco Life Technologies, Rockville, MD, USA).

***Enzyme-linked immunosorbent assay (ELISA)***

Briefly, 1×10^5^ cells/ml from different experimental groups were plated on confocal Petri dishes in serum-containing media for 24 h. The cells were then incubated in conditioned media alone, 6-OHDA (100 μM) alone, 6-OHDA (100 μM) + *Cst3* knockdown or overexpression of CYS C. After 48 h, the supernatants were collected. TNF-α, IL-1β, VEGF levels in supernatants were determined with the TNF-α, IL-1β, VEGF ELISA kits (R&D Systems) according to the manufacturer's protocols, respectively.

***Protein Extraction, Subcellular Fractionation, and Western Blot Analysis in 6-OHDA-lesioned PC12 cells***

Western blot analysis with appropriately diluted antibodies of :rabbit anti-CYS C (Abcam; 1:1000),rabbit anti-NURR1 (Sigma; 1:1000), rabbit anti-NUR77 (Santa Cruz Biotechnology; 1:500), rabbit anti-tyrosine hydroxylase (TH) (Cell Signaling Technology; 1:1000), rabbit anti-dopamine transporter (DAT) (Santa Cruz Biotechnology; 1:500), rabbit anti-SNCA (Cell Signaling Technology; 1:1000), rabbit anti-Ser129-phosphorylated SNCA (Cell Signaling Technology; 1:1000), rabbit anti-ERK/p-ERK (Cell Signaling Technology; 1:1000), rabbit anti-PKC/p-PKC (Cell Signaling Technology; 1:1000), rabbit anti-PKC-α/p-PKC-α (Cell Signaling Technology; 1:1000), rabbit anti-PKC-β/p-PKC-β(Cell Signaling Technology; 1:1000), rabbit anti-PKC-ζ/p-PKC-ζ(Cell Signaling Technology; 1:1000),rabbitanti-LC3B-I/II (Sigma; 1:1000), rabbit anti-LC3B-II (Cell Signaling Technology; 1:1000), rabbit anti-SQSTM1 (Abcam; 1:1000), mouse anti-VEGF (Abcam; 1:1000), rabbit anti- LMNB1 (Cell Signaling Technology; 1:1000), rabbit anti-TUBA (Cell Signaling Technology; 1:1000) and rabbit anti-ACTB (Cell Signaling Technology; 1:1000). For inhibition of the extracellular signal-regulated protein kinases (ERK), protein kinase C (PKC) and vascular endothelial growth factor receptor 2 (VEGFR2 or KDR), their inhibitors, i.e, U0126 (10 μM), GF109203 (3 μM), and SU5614 (5 μM) were respectively pre-incubated for 30 minutes before 6-OHDA addition.

For the protein extractions prepared from the cytosolic and nuclear fractions, the cells were washed 3 times with cold PBS and were harvested by centrifugation at 1100 rpm for 10 min. The cell pellet was resuspended in 3 pellet volumes of cold buffer A (20 mM HEPES, pH 7.0, 0.15 mM ethylene diamine tetraacetic acid[EDTA], 0.015 mM ethylene glycol tetraacetic acid[EGTA], 10 mM potassium chloride[KCl], 1% Nonidet P-40, 1 mM phenylmethylsulfonyl fluoride, 20 mM sodium fluoride[NaF], 1 mM sodium pyrophosphate, 1 mM sodium orthovanadate, 1 μg/ml leupeptin) and was incubated on ice for 30 min. Then, the homogenate was centrifuged at 500 x*g* for 5 min. The supernatants were taken as the cytosolic fraction. The nuclear pellet was re-suspended in 5 volumes of cold buffer B (10 mM HEPES, pH 8.0, 0.1 mM EDTA, 0.1 mM sodium chloride[NaCl], 25% glycerol, 1 mM phenylmethylsulfonyl fluoride, 20 mM NaF, 1 mM sodium pyrophosphate, 1 mM sodium orthovanadate, 1 μg/ml leupeptin). After centrifugation under the same conditions indicated above, the nuclei were resuspended in loading buffer containing 0.5% sodium dodecyl sulfate (SDS). The cytosolic and nuclear fractions were resolved using sodium dodecyl sulfate-polyacrylamide gel electrophoresis (SDS-PAGE) and were immunoblotted with the indicated antibodies.

***Immunofluorescence in 6-OHDA-lesioned PC12 cells***

Briefly, after three washes in PBS, fixation and permeabilization of the cells were conducted with 4% paraformaldehyde in PBS for 15 min at room temperature, and the cells were rinsed with PBS and then incubated with PBS-T (0.3% Triton X-100 in PBS) for 15 min. After three more washes with PBS, the cells were incubated with blocking buffer (PBS, 3% bovine serum albumin[BSA])for 60 min at room temperature. The following primary antibodies were used: rabbit anti-NURR1 (Sigma; 1:200), rat anti-DAT (Millipore; 1:5000), rabbit anti-TH (Millipore; 1:2000), goat anti-CYS C (Santa; 1:100), mouse anti-VEGF (Abcam; 1:200), rabbit anti-Ser129-phosphorylated SNCA (Sigma; 1:200), rabbit anti-SNCA (Cell Signaling Technology; 1:800), rabbit anti-cleaved CASP3 (Cell Signaling Technology; 1:800), rabbit anti-LC3B (Sigma; 1:100), mouse anti-SQSTM1 (Abcam; 1:100) and 4',6-diamidino-2-phenylindole dihydrochloride (DAPI) (300 nmol/L, Invitrogen, Carlsbad, CA, USA). The specimens were incubated overnight at 4°Cwith the primary antibodies in PBS containing 3% BSA. For immunofluorescence, on the following day, after additional rinsing with PBS, the samples were incubated with fluorescent-labeled secondary antibodies (Alexa 488-, Alexa 568-, Alexa 555- or Alexa 647-labeled IgG; Invitrogen) in PBS containing 3% BSA for 60 min at room temperature. After rinsing with PBS, DAPI (300 nmol/L, Invitrogen, Carlsbad, CA, USA) was used for counterstaining for 3-5 min. The confocal analysis was performed using a Zeiss LSM 710/Meta Station (Carl Zeiss) equipped with a digital camera (Hamamatsu, Japan) and operated by QED imaging software.

***TdT-mediated dUTP-X nick end labeling Assay (TUNEL)***

Apoptosis in cells sample was assessed using a TUNEL Assay Kit (Roche) according to the manufacturer's instructions. PC12 cells were analyzed after the corresponding treatment periods. Cells were conducted with 4% paraformaldehyde in PBS for 15 min at room temperature, and the cells were rinsed with PBS and then incubated with PBS-T (0.3% Triton X-100 in PBS) for 15 min. After three more washes with PBS, the cells were incubated with blocking buffer (PBS, 3% BSA) for 60 min at room temperature. TUNEL labeling (TdT-mediated dUTP nick-end labeling) was performed and the DNA strand breaks were labeled with fluorescein for visualization. Slides were examined using a fluorescein isothiocyanate (FITC) filter on a Zeiss LSM 710/Meta Station (Carl Zeiss) equipped with a digital camera (Hamamatsu, Japan) and operated by QED imaging software.

***Statistical Analysis***

The strength of the association for correlation coefficients was interpreted as follows: ≤ 0.19, negligible; 0.20 to 0.39, weak; 0.40 to 0.59, moderate; 0.60 to 0.79, strong; and ≥ 0.80, very strong . The cutoff, AUC, sensitivity, and specificity were used to assess discriminative ability.

**Supplementary Figure 1. Knockdown of *Cst3* promotes cellular apoptosis of DAergic PC12 cells.**

(***A-D***) Knockdown of *Cst3* by siRNAs promoted 6-OHDA-induced apoptosis of PC12 cells. PC12 cells were nontransfected or transfected with NC or siRNAs for 48 hours and then were incubated with 100 μM 6-OHDA for another 24 hours, followed by flow cytometry with double staining annexin V-FITC and PI for the (***A***) blank group, (***B***) NC + 6-OHDA, (***C***) siRNA-1 + 6-OHDA and (***D***) siRNA-2 + 6-OHDA.

**Supplementary Figure 1.**
